# Supplementary figures and images for: Echocardiographic Changes in Dogs with Stage B2 Myxomatous Mitral Valve Disease Treated with Pimobendan Monotherapy
Source: Vet Sci. 2024 Nov 25;11(12):594. doi: 10.3390/vetsci11120594 (PMC11680358; doi:10.3390/vetsci11120594)

# Supplementary Figures

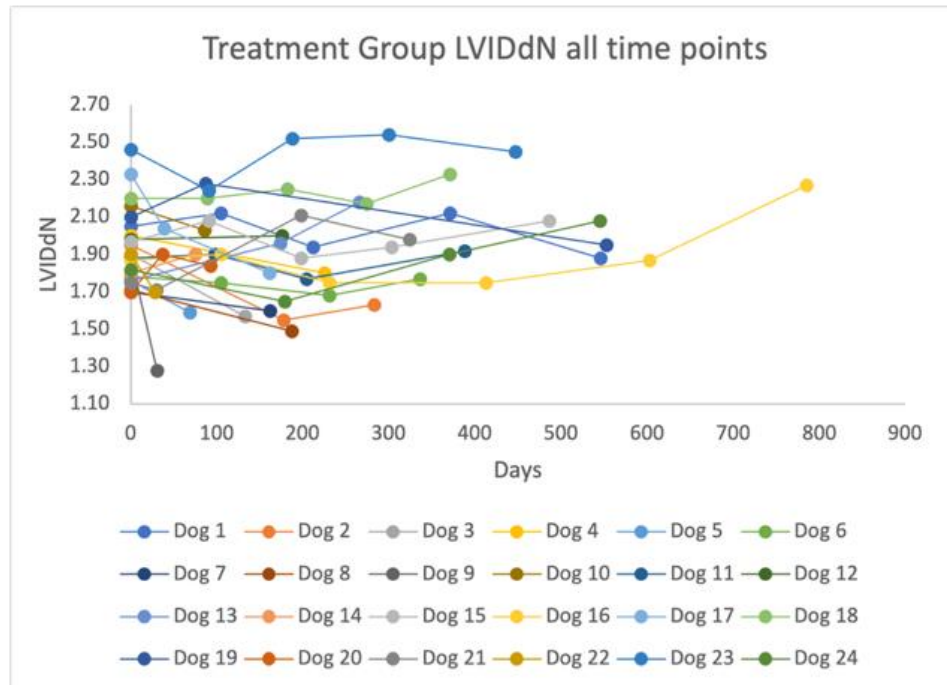

Supplementary Figure S1

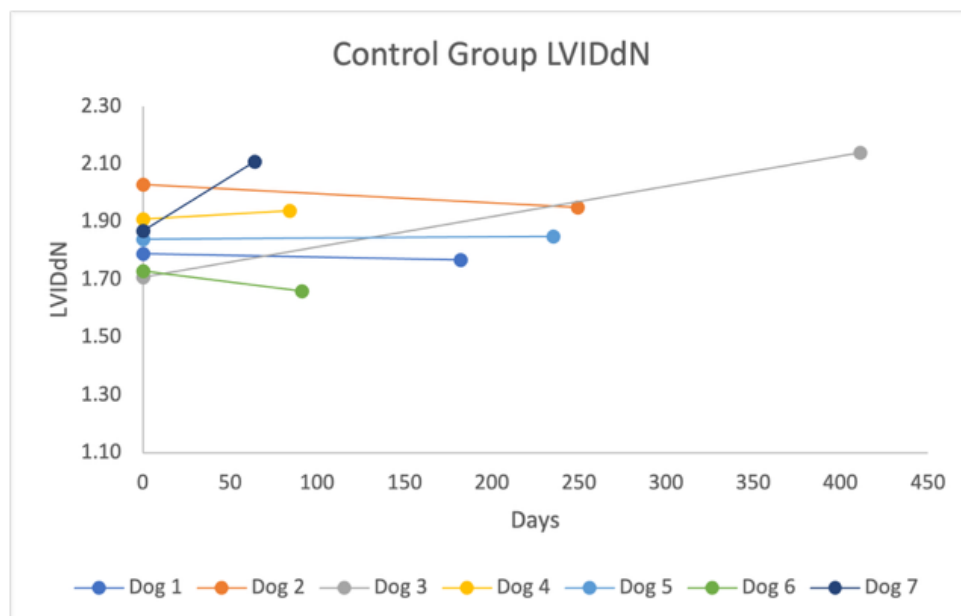

Supplementary Figure S2

Supplement: Supplementary file 1 [file vetsci-11-00594-s001.zip › vetsci-3256944-Supplementary Figures.pdf]
